# Effects of providing sensory attractants to suckling pigs during lactation and after weaning on post-weaning growth performance

**DOI:** 10.1093/tas/txac170

**Published:** 2022-12-30

**Authors:** Madie R Wensley, Mike D Tokach, Jason C Woodworth, Robert D Goodband, Joel M DeRouchey, Jordan T Gebhardt, Denny McKilligan, Nathan Upah

**Affiliations:** Department of Animal Sciences and Industry, College of Agriculture, Kansas State University, Manhattan, KS, 66506-0201, USA; Department of Animal Sciences and Industry, College of Agriculture, Kansas State University, Manhattan, KS, 66506-0201, USA; Department of Animal Sciences and Industry, College of Agriculture, Kansas State University, Manhattan, KS, 66506-0201, USA; Department of Animal Sciences and Industry, College of Agriculture, Kansas State University, Manhattan, KS, 66506-0201, USA; Department of Animal Sciences and Industry, College of Agriculture, Kansas State University, Manhattan, KS, 66506-0201, USA; Department of Diagnostic Medicine/Pathobiology, College of Veterinary Medicine, Kansas State University, Manhattan, KS, 66506-0201, USA; TechMix, LLC., Stewart, MN, 55057, USA; TechMix, LLC., Stewart, MN, 55057, USA

**Keywords:** body weight loss, enrichment, growth performance, nursery pig, sensory attractant, weaning

## Abstract

Three experiments were conducted to determine the effect of sensory attractants pre- and post-weaning on the growth performance of pigs after weaning. For each experiment, treatments were arranged as a 2 × 2 × 2 factorial with main effects of pre-weaning application (without or with), post-weaning application (without or with), and body weight category (representing the lightest or heaviest 50% of the population). In Exp. 1, 356 nursery pigs (initially 5.7 kg) were used in a 28-d trial with enrichment cubes used as the sensory attractant. A greater percentage of heavy pigs (*P* *=* 0.007) or pigs offered enrichment cubes pre-weaning (*P* *=* 0.044) lost BW from weaning to d 3 compared to light pigs or pigs not offered enrichment cubes pre-weaning. From weaning to d 7, a greater percentage of pigs lost weight when not offered cubes post-weaning (*P* *=* 0.002) compared to pigs offered cubes post-weaning. In Exp. 2, 355 nursery pigs (initially 5.6 kg) were used in a 29-d trial with a powder used as the sensory attractant. Providing a powder attractant both pre- and post-weaning reduced the percentage of pigs that lost weight from weaning to d 3 as compared with providing a powder either pre- or post-weaning only (interaction, *P* < 0.05). In Exp. 3, 355 nursery pigs (initially 5.9 kg) were used in a 24-d trial with a liquid spray used as the sensory attractant. A greater percentage of heavy pigs that did not receive liquid attractant lost weight from weaning to d 3, whereas a greater percentage of light pigs lost weight when they received liquid attractant only pre-weaning (three-way interaction; *P* *=* 0.016). Across all three experiments, sensory attractant application had limited effects on the growth performance of pigs after weaning; however, varying responses were observed for the percentage of pigs that lost weight in the first 3 to 7 d immediately post-weaning. In summary, environmental enrichment with cubes (Exp. 1) appears to have the greatest effect when applied post-weaning whereas flavor attractants (Exp. 2 and 3) appear to have the greatest effect when applied both pre- and post-weaning.

## Teaser

Sensory attractants may encourage early development of feeding behavior, therefore decreasing the percentage of pigs that lose weight after weaning, as measured by the percentage of pigs that were still below weaning weight 3 or 7 d post-weaning.

## INTRODUCTION

At the time of weaning, pigs generally have limited experience with solid feed. This often results in low feed intake and body weight (BW) gain post-weaning (Bruininx et al., 2001). Low feed intake has been shown to disrupt the natural development of the gastrointestinal tract, leading to losses in intestinal barrier structure and function (McCracken et al., 1999; Moeser et al., 2017). A review by Wensley et al. (2021a, 2021b) evaluated different feeding and management strategies to minimize the negative consequences of weaning. Among the strategies reported, behavioral development through sensory learning is thought to elicit early feed intake. Sensory learning encourages pigs to use their senses to explore the environment around them. Using sensory learning in combination with feeding may offer an opportunity to ease the weaning transition by training pigs to respond to familiar sensory stimuli. Therefore, it’s suggested that providing flavor, odor, or environmental stimuli both before and after weaning may improve feed intake post-weaning because of sensory association (Oostindejer, 2014). However, little data is available that demonstrates meaningful improvements in performance. Likewise, inconsistent results have been reported when adding flavor enhancers to only starter diets to increase feed acceptance after weaning (Oostindjer et al., 2010a; Blavi et al., 2016). Hence for these experiments, we hypothesized that applying either an enrichment object, powder, or liquid sensory attractant pre- and post-weaning would evoke the greatest post-weaning response as it would provide pigs familiar stimuli, consequently reducing feed neophobia. Likewise, it was hypothesized that administering sensory attractants pre-weaning, but not post-weaning, would be detrimental, resulting in decreased feed intake and increased BW loss immediately after weaning. Thus, the objective of these studies was to determine the effect of sensory attractants pre- and post-weaning on the growth performance of pigs after weaning.

## MATERIALS AND METHODS

The Kansas State University Institutional Animal Care and Use Committee approved the protocols used in these experiments. Each experiment was conducted at the Kansas State University Swine Teaching and Research Center in Manhattan, KS. In the nursery, each pen (1.2 × 1.2 m) contained a 4-hole, dry self-feeder, and nipple waterer for ad libitum access to feed and water. Pigs were weighed and feed disappearance measured on d 3, 7, 14, and 24 or 25 after weaning to determine ADG, ADFI, and G:F. The percentage of pigs that lost weight (pigs that were still below weaning weight) from d 0 to 3 and d 0 to 7 post-weaning was also determined.

### Experiment 1

A total of 28 litters (241 × 600, DNA, Columbus, NE) were used. Sows were fed a common lactation diet throughout the experimental period. Four days prior to weaning, pigs were weighed, and litters allotted to 1 of 2 treatment groups in a randomized complete block design based on sow parity and average piglet BW. Treatments consisted of a negative control (no enrichment cubes) or an enrichment cube treatment, in which approximately 100 g of cubes (7 cubes) were provided to litters once daily (AM) for 4 d pre-weaning. Cubes were placed directly on the floor in the center of farrowing stalls on the opposite side of the heat lamp.

At weaning (approximately 20 d of age), 356 pigs (initially 5.7 kg) were weighed and evenly divided into light or heavy BW categories within pre-weaning treatment (representing the lightest or heaviest 50% of the population) and allotted to nursery pen. Each pen was randomized to 1 of 4 treatments with 4 or 5 pigs per pen and 18 replications per combination of pre- and post-weaning treatment. Body weight category was equally distributed across treatment groups. Treatments were arranged in a 2 × 2 × 2 factorial with main effects of pre-weaning application (without or with enrichment cubes), post-weaning application (without or with enrichment cubes), and BW category [light (2.4–5.7 kg) or heavy (5.8–9.0 kg)]. Pens of pigs assigned to the enrichment cube treatment group were offered approximately 100 g of cubes (7 cubes) once daily (AM) in the pan of the fence-line feeder for 3 d after weaning.

**Figure 1.**
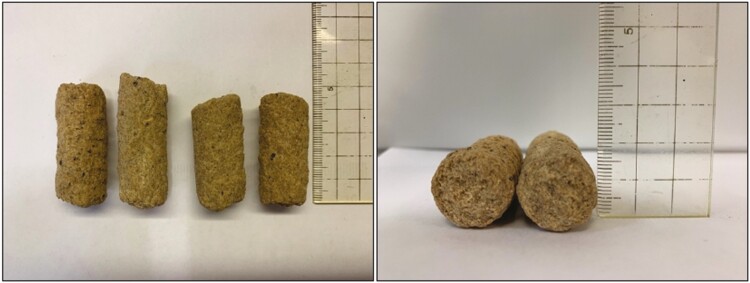
Enrichment cubes ranged in size from 28 to 51 mm in length and 20 mm diameter and were manufactured by Form-A-Feed (Stewart, MN).

Wean pigs are attracted to materials that are odorous, destructible, and chewable (Van deWeerd et al., 2003). Young pigs also prefer pellets with a large diameter (12 mm) compared to smaller pellets (van den Brand et al., 2014). Therefore, large destructible cubes were used as the sensory attractant for this experiment. Cubes ranged in size from 28 to 51 mm in length and 20 mm in diameter and were manufactured by Form-A-Feed in Stewart, MN (Figure 1). Cubes were not intended to be a complete diet replacement. Instead, cubes were designed to stimulate the pig’s olfactory senses through the inclusion of aromatic polyphenols (Blavi et al., 2016). Other ingredients used in cube formulation included soy hulls, soybean meal, wheat midds, and digestible sugars. Together, these ingredients provided pigs a large object that allowed for oral-nasal interaction, such that pigs could play with, chew on, and slowly begin to destroy the cubes over time. In addition to the enrichment cubes, all pigs received 3.5 mm pelleted corn-soybean meal based starter diets for the 24-d feeding program after weaning, fed in 2 phases with phase 1 fed from d 0 to 7 and phase 2 fed from d 7 to 24.

### Experiment 2

A total of 28 litters (241 × 600, DNA, Columbus, NE) were used. Sows were fed a common lactation diet throughout the experimental period. Four days prior to weaning, pigs were weighed and litters were allotted to 1 of 2 treatment groups in a randomized complete block design based on sow parity and average piglet BW. Treatments consisted of a control (without powder) or a treatment with powder, in which approximately 90 g of sensory attractant powder, divided into 2 feedings (AM and PM), were provided in the pan of rotary feeders for 4 d pre-weaning. Litters assigned to the control were not exposed to rotary feeders.

At weaning (approximately 20 d of age), 355 pigs (initially 5.6 kg) were weighed and evenly divided into light or heavy BW categories within pre-weaning treatment (representing the lightest or heaviest 50% of the population) and allotted to nursery pens. Each pen was randomized to 1 of 4 treatments with 5 or 6 pigs per pen and 15 replications per combination of pre- and post-weaning treatment. Body weight category was equally distributed across treatment groups. Treatments were arranged in a 2 × 2 × 2 factorial with main effects of pre-weaning application (without or with powder), post-weaning application (without or with powder), and BW category [light (2.6–5.5 kg) or heavy (5.7–8.1 kg)]. Pens of pigs assigned to the powder treatment group were offered approximately 45 g of sensory attractant powder per day, divided into 2 feedings (AM and PM) top-dressed on feed in the feeder pan for 2 d post-weaning. On d 3, approximately 45 g of feed (taken from the back of the feeder), divided into 2 feedings (AM and PM), was top-dressed on feed in the feeder pan to mimic powder application. This was done to maximize the behavioral response of top dressing the sensory attractant powder.

Baby Pig Restart APF (TechMix LLC; Stewart, MN) was used as the sensory attractant for this experiment. Baby Pig Restart APF is a palatable powder designed to entice pigs to eat dry feed. Because smell is experienced prior to consumption, aroma is an important first introduction to feed intake (Frederick and van Heugten, 2006). Hence, ingredients within Baby Pig Restart APF such as digestible sugars, aromatic polyphenols (Blavi et al., 2016), and amino acids with umami attributes (Tedo et al., 2017) provide both oral and nasal stimuli. Baby Pig Restart APF also provides additional nutrients as it is formulated to contain 19.5% crude protein and 2.75% crude fat. Because powder application is not intended to replace complete feed, all pigs received common 3.5 mm pelleted corn-soybean meal based phase 1 and 2 diets throughout the duration of the 25-d trial. Phase 1 diet was provided from d 0 to 7 and phase 2 diet from d 7 to 25 post-weaning.

### Experiment 3

A total of 28 litters (241 × 600, DNA, Columbus, NE) were used. Sows were fed a common lactation diet throughout the experimental period. Within 24 h of farrowing, litters of pigs were weighed and allotted to 1 of 2 treatment groups in a completely randomized design based on farrowing date. Sow parity, average piglet BW, and average litter size were balanced across treatment groups. Treatments consisted of a control (without liquid sensory attractant) or a treatment with liquid sensory attractant, in which approximately 89 ml of liquid sensory attractant per day, divided into two applications (AM and PM), was sprayed on the underline of sows for 2 d beginning the morning after farrowing. Treatment application was later resumed for 2 d pre-weaning. In total, pigs received liquid sensory attractant for 4 d pre-weaning.

At weaning (approximately 20 d of age), 355 pigs (initially 5.9 kg) were weighed and evenly divided into light or heavy BW categories within pre-weaning treatment (representing the lightest or heaviest 50% of the population) and allotted to nursery pens. Each pen was randomized to 1 of 4 treatments with 5 or 6 pigs per pen and 15 replications per combination of pre- and post-weaning treatment. Body weight category was equally distributed across treatment groups. Treatments were arranged in a 2 × 2 × 2 factorial with main effect of pre-weaning application (without or with spray), post-weaning application (without or with spray), and BW category [light (2.6–5.8 kg) or heavy (6.0–8.9 kg)]. Pens of pigs assigned to the liquid attractant treatment group were offered approximately 59 ml of liquid per day, divided into three applications (7:00, 12:00, and 17:00), sprayed on the feed in the feeder pan. Treatment application continued for 3 d post-weaning.

BlueLite Pro2Lyte (TechMix LLC; Stewart, MN) was used as the sensory attractant for this experiment. BlueLite Pro2Lyte is a dry powdered electrolyte that when mixed with water according to label instructions creates an isotonic solution. Like Baby Pig Restart AFP used in Exp. 2, BlueLite Pro2Lyte contains digestible sugars, aromatic polyphenols, and amino acids with umami attributes. These ingredients have been shown to be highly palatable and stimulate the olfactory senses of the pig (Blavi et al., 2016; Tedo et al., 2017). Pigs were fed common 3.5 mm pelleted corn-soybean meal based phase 1 and 2 diets throughout the duration of the 24-d trial. Phase 1 diet was provided from d 0 to 7, and phase 2 diet from d 7 to 24.

### Data Analysis

In Exp. 1 and 2, pre-weaning data were analyzed as a randomized complete block design with litter as the experimental unit. Treatment was considered a fixed effect and block was included in the model as a random effect which incorporated both sow parity and average piglet BW. In Exp. 3, pre-weaning data were analyzed as a completely randomized design with litter as the experimental unit. Treatment was considered a fixed effect and parity was included in the model as a random effect. Total born count was analyzed using a Poisson model.

Post-weaning data for each experiment were analyzed as a factorial with main effect of: 1) pre-weaning treatment (without or with); 2) post-weaning treatment (without or with); and 3) BW category (light or heavy). Pen was considered the experimental unit. Least square means were applied to estimate the interactive and main effects. A binomial model was used to determine the percentage of pigs within a pen that lost weight from d 0 to 3 and d 0 to 7 post-weaning using a logit link function. All statistical models were fit using the GLIMMIX procedure of SAS v. 9.4 (SAS Institute, Inc., Cary, NC). Results were considered significant at *P* ≤ 0.05.

## RESULTS

### Experiment 1

As expected, providing enrichment cubes to litters pre-weaning did not influence piglet weaning weights (*P* = 0.976; without = 5.7 kg; with = 5.7 kg). Interestingly, several three-way interactions were observed after weaning for pre- and post-weaning enrichment cube application and BW category (Table 1; interactive means are only shown for the significant response variables). Heavy weight pigs that were provided enrichment cubes before weaning, but not after, grew slower and ate less feed from d 7 to 14, 14 to 24, and 0 to 24 post-weaning compared to heavy pigs that were provided enrichment cubes before and after weaning. The opposite effect was observed for light weight pigs (*P* < 0.05). This led to increased final BW in heavy weight pigs provided enrichment cubes both before and after weaning and decreased final BW (*P* < 0.05) in light weight pigs provided enrichment cubes before weaning but not after weaning, with all other treatment combinations intermediate. If pigs did not receive enrichment cubes before weaning, providing the cubes after weaning had no impact on performance in either light or heavy pigs. No differences in growth performance (*P* > 0.10) after weaning were observed for the overall interaction between pre × post-weaning treatment, as well as the overall interaction between post-weaning treatment × BW category (*P* > 0.05; data not shown). No differences in overall ADG or G:F (*P* > 0.10) were observed for the interaction between pre-weaning treatment × BW category.

**Table 1.** Body weight category × pre-weaning enrichment cube × post-weaning enrichment cube interaction on nursery pig growth performance, Exp. 1^1^

Item	BW category								SEM	*P* *=* interaction^2^
	Light				Heavy					
	Pre-wean cube									
	Without		With		Without		With			
	Post-wean cube									
	Without	With	Without	With	Without	With	Without	With		
Post-wean BW, kg										
d 14	6.6^c^	6.4^c^	6.8^c^	6.5^c^	9.0^a^	8.9^a,b^	8.5^b^	9.0^a^	0.14	0.046
d 24	10.3^c,d^	10.2^d^	11.0^c^	10.4^cd^	13.9^a,b^	13.5^a,b^	13.2^b^	14.0^a^	0.27	0.034
d 7 to 14										
ADG, g	210^c,d,e^	200^d,e^	229^b,c,d^	185^e^	282^a^	246^a,b,c^	213^c,d,e^	256^a,b^	13.8	0.005
ADFI, g	225^d^	232^c,d^	254^c,d^	231^c,d^	324^a^	312^a^	265^b,c^	296^a,b^	12.9	0.044
d 14 to 24										
ADG, g	375^d^	374^d^	429^b,c^	387^c,d^	488^a^	467^a,b^	472^a,b^	504^a^	16.9	0.047
ADFI, g	512^d^	526^d^	553^c,d^	529^d^	662^a^	625^a,b^	604^b,c^	652^a,b^	19.1	0.026
d 0 to 24										
ADG, g	233^d,e^	228^e^	262^c,d^	237^d,e^	300^a,b^	284^a,b,c^	275^b,c^	304^a^	10.4	0.028
ADFI, g	310^d^	317^d^	336^c,d^	324^d^	409^a^	392^a,b^	363^b,c^	398^a^	12.2	0.042

^a,b,c^Means lacking common superscript differ, *P* < 0.05.

^1^For the pre-weaning portion of the experiment, a total of 28 litters (241 × 600, DNA, Columbus, NE) were used. Treatments consisted of a negative control (no enrichment cubes) or an enrichment cube treatment, in which approximately 100 g of cubes (7 cubes) were provided to litters once daily (AM) on the floor of farrowing stalls for 4 d prior to weaning. For the post-weaning portion of the experiment, a total of 356 pigs were weaned and used in a 24-d growth trial with 4 or 5 pigs per pen and 18 replicates per treatment. Treatments were arranged in a 2 × 2 × 2 factorial with main effects of pre-weaning treatment (without or with enrichment cubes), post-weaning treatment (without or with enrichment cubes), and BW category (light or heavy). Pens of pigs assigned to the enrichment cube treatment group were offered approximately 100 g of cubes (7 cubes) once daily (AM) in feeder pans for 3 d after weaning. For ease of interpretation, only the significant response variables are shown for the three-way interactions between BW category and pre- and post-weaning treatment.

^2^In addition to the reported three-way interaction *P*-values, a BW category × post-weaning enrichment cube interaction was observed in d 14 BW (*P* = 0.043). A BW category × pre-weaning enrichment cube interaction was observed in d 7 to 14 ADFI (*P* = 0.006) and d 0 to 24 ADFI (*P* = 0.037).

For the main effect of pre-weaning cube application, no overall differences were observed on post-weaning ADG or ADFI (*P* > 0.10; Table 2). However, pigs offered enrichment cubes prior to weaning had improved overall post-weaning G:F (*P* *=* 0.017) compared to pigs not offered enrichment cubes. This response was also observed from d 0 to 7 (*P* *=* 0.064) and 14 to 24 (*P* *=* 0.009). For the main effect of post-weaning cube application, no effect on the ADG or ADFI (*P* > 0.10) of pigs after weaning was observed. There was a tendency for pigs offered enrichment cubes post-weaning to have poorer G:F (*P* *=* 0.078) compared to pigs not offered enrichment cubes. For the main effect of BW category, heavy weight pigs had improved overall ADG (*P* *<* 0.001) and ADFI (*P* *<* 0.001) compared to light weight pigs. No evidence for difference in G:F (*P* > 0.10) were observed.

**Table 2.** Main effect of pre-weaning enrichment cube, post-weaning enrichment cube, and BW category on the growth performance of nursery pigs, Exp. 1^1^

Item	Pre-wean cube		SEM	*P* =	Post-wean cube		SEM	*P* =	BW category		SEM	*P* =
	Without	With			Without	With			Light	Heavy		
Post-wean BW, kg												
d 0	5.7	5.7	0.02	0.935	5.7	5.7	0.02	0.688	4.7	6.7	0.02	< 0.001
d 3	5.7	5.7	0.03	0.486	5.6	5.7	0.03	0.138	4.7	6.6	0.03	< 0.001
d 7	6.1	6.1	0.04	0.145	6.1	6.1	0.04	0.257	5.1	7.1	0.04	< 0.001
d 14	7.7	7.7	0.07	0.932	7.7	7.7	0.07	0.891	6.6	8.8	0.07	< 0.001
d 24	12.0	12.1	0.13	0.313	12.1	12.0	0.12	0.699	10.4	13.7	0.13	< 0.001
Pigs that lost BW after weaning, %^2^												
d 3	38.4	49.5	3.91	0.044	48.7	39.1	3.89	0.081	36.5	51.5	3.91	0.007
d 7	8.1	7.7	2.51	0.907	15.5	3.8	2.76	0.002	10.1	6.1	2.43	0.246
d 0 to 7												
ADG, g	54	65	5.4	0.170	54	64	5.4	0.181	59	60	5.4	0.904
ADFI, g	120	122	4.4	0.849	116	126	4.4	0.114	111	131	4.4	0.003
G:F, g/kg	412	511	37.1	0.064	425	498	37.1	0.171	483	441	37.1	0.426
d 7 to 14												
ADG, g	235	221	6.9	0.168	233	222	6.9	0.242	206	249	6.9	< 0.001
ADFI, g	273	262	6.4	0.210	267	268	6.4	0.921	235	299	6.4	< 0.001
G:F, g/kg	869	841	18.3	0.295	880	830	18.3	0.054	877	833	18.3	0.090
d 14 to 24												
ADG, g	425	448	8.4	0.061	441	432	8.4	0.468	391	482	8.4	< 0.001
ADFI, g	581	585	9.5	0.799	583	583	9.5	0.999	530	636	9.5	< 0.001
G:F, g/kg	731	766	9.3	0.009	756	741	9.3	0.279	738	759	9.3	0.110
d 0 to 24												
ADG, g	261	270	5.2	0.257	267	264	5.2	0.604	240	291	5.2	< 0.001
ADFI, g	357	355	6.1	0.839	354	358	6.1	0.697	322	390	6.1	< 0.001
G:F, g/kg	733	759	7.5	0.017	755	736	7.5	0.078	745	746	7.5	0.880

^1^For the pre-weaning portion of the experiment, a total of 28 litters (DNA 241 × 600, Columbus, NE) were used. Treatments consisted of a negative control (no enrichment cubes) or an enrichment cube treatment, in which approximately 100 g of cubes (7 cubes) were provided to litters once daily (AM) on the floor of farrowing stalls for 4 d prior to weaning. For the post-weaning portion of the experiment, a total of 356 pigs were weaned and used in a 24-d growth trial with 4 or 5 pigs per pen and 18 replicates per treatment. Treatments were arranged in a 2 × 2 × 2 factorial with main effects of pre-weaning treatment (without or with enrichment cubes), post-weaning treatment (without or with enrichment cubes), and BW category (light or heavy). Pens of pigs assigned to the enrichment cube treatment group were offered approximately 100 g of cubes (7 cubes) once daily (AM) in feeder pans for 3 d after weaning.

^2^Represents the percentage of pigs that were still below weaning weight 3 or 7 d post-weaning.

While limited effects of enrichment cubes were observed on the growth performance of pigs after weaning, the true value of enrichment cubes may be in the percentage of pigs that lost weight 3 or 7 d post-weaning, as measured by the percentage of pigs that were still below weaning weight. No evidence for differences were observed in the three-way interaction or any of the two-way interactions for piglet BW loss (*P* > 0.05). For the main effect of pre-weaning cube application, a greater percentage of pigs lost weight (*P* *=* 0.044) from d 0 to 3 post-weaning when offered enrichment cubes prior to weaning compared to no cubes. Conversely, for the main effect of post-weaning cube application, the percentage of pigs that lost weight from d 0 to 7 post-weaning was reduced by 11.7 percentage points (*P* *=* 0.002) when pigs were offered enrichment cubes compared to no cubes. There was no evidence for differences for the main effect of BW category (*P* > 0.10) at d 7 post-weaning; however, the percentage of pigs that lost weight 3 d post-weaning was 15 percentage points greater (*P* = 0.007) for the heavy weight pig population than the lightweight pig population. This suggests heavy weight pigs have an easier time recovering from the initial weight loss that occurs after weaning.

### Experiment 2

Pre-weaning powder application did not influence piglet weaning weight (*P* = 0.485; without = 5.5 kg; with = 5.6 kg). Post-weaning, no three-way interactions (*P* > 0.10) between pre- and post-weaning powder application and BW category were observed (data not shown). Likewise, no overall interactions (*P* > 0.10) between pre × post-weaning treatment were observed, as well as the overall interaction (*P* > 0.05) between post-weaning treatment × BW category. However, several interactions between pre-weaning powder application × BW category were observed (Table 3; interactive means are only shown for the significant response variables). Pre-weaning powder application did not affect post-weaning ADFI in the lightweight pig population, but increased feed intake in the heavy weight pig population (*P* *=* 0.027). Because ADFI did not impact ADG, differences in G:F (*P* *=* 0.013) were also observed. Powder application pre-weaning improved overall G:F in the light weight pig population compared to the heavy weight pig population, with the other treatment combinations intermediate. A similar response in ADFI and G:F were also observed from d 14 to 25. No differences were observed in ADG throughout (*P* > 0.10).

**Table 3.** Body weight category × pre-weaning sensory attractant powder interaction on the growth performance of nursery pigs, Exp. 2^1^

Item	BW category				SEM	*P* = ^2^ Interaction
	Light		Heavy			
	Pre-wean powder					
	Without	With	Without	With		
d 0 to 7						
ADFI, g	71^b^	68^b^	68^b^	87^a^	4.3	0.014
d 14 to 25						
ADFI, g	430^b^	397^b^	510^a^	551^a^	16.7	0.030
G:F, g/kg	731^a,b^	783^a^	768^a,b^	720^b^	19.2	0.012
d 0 to 25						
ADFI, g	267^c^	254^c^	316^b^	346^a^	9.3	0.027
G:F, g/kg	677^a,b^	710^a^	707^a,b^	671^b^	13.3	0.013

^a,b,c^Means lacking common superscript differ, *P* < 0.05.

^1^For the pre-weaning portion of the experiment, a total of 28 litters (241 × 600, DNA, Columbus, NE) were used during one lactation period. Treatments consisted of a negative control (without powder) or a treatment with powder, in which approximately 45 g of sensory attractant powder were provided to litters of pigs twice daily (AM and PM) in the pan of rotary creep feeders for 4-d prior to weaning. For the post-weaning portion of the experiment, a total of 355 pigs were weaned and used in a 25-d growth trial with 5 or 6 pigs per pen and 15 replicates per treatment. Treatments were arranged in a 2 × 2 × 2 factorial with main effects of pre-weaning treatment (without or with powder); post-weaning treatment (without or with powder); and BW category (light or heavy). Pens of pigs assigned to the powder treatment group were offered approximately 22.5 g of sensory attractant powder twice daily (AM and PM), that was top-dressed on feed in feeder pans for 2 d after weaning. On d 3 approximately 22.5 g of feed (taken from the back of the feeder) was top-dressed on feed in the feeder pans twice daily to mimic powder application.

^2^No three-way interactions between pre- and post-weaning treatment and BW category were observed for growth performance after weaning (*P* > 0.10). Likewise, no post-weaning treatment × BW category interactions were observed (*P* > 0.10). For ease of interpretation, only the significant response variables are shown for the interaction between pre-weaning treatment × BW category.

For the main effect of pre-weaning powder application, no differences were observed on post-weaning performance (*P* > 0.05; Table 4). Likewise, no differences were observed for the main effect of post-weaning powder application (*P* > 0.10). For the main effect of BW category, no differences were observed in growth performance the first 7 d post-weaning (*P* > 0.05). This suggests that heavy weight and light weight pigs all struggle to make the weaning transition, regardless of starting BW. However, overall (d 0 to 25), heavy weight pigs had increased ADG (*P* *<* 0.001) and ADFI (*P* < 0.001) compared to light weight pigs, with no evidence for differences observed in G:F (*P* > 0.10).

**Table 4.** Main effect of pre-weaning sensory attractant powder, post-weaning sensory attractant powder, and BW category on the growth performance of nursery pigs, Exp. 2^1^

Item	Pre-wean powder		SEM	*P* =	Post-wean powder		SEM	*P* =	BW category		SEM	*P* =
	Without	With			Without	With			Light	Heavy		
Post-wean BW, kg												
d 0	5.6	5.5	0.03	0.593	5.6	5.5	0.03	0.714	4.7	6.4	0.03	<0.001
d 3	5.2	5.3	0.03	0.494	5.2	5.3	0.03	0.415	4.4	6.1	0.03	<0.001
d 7	5.6	5.7	0.04	0.428	5.6	5.7	0.04	0.356	4.8	6.5	0.04	<0.001
d 14	6.8	6.8	0.07	0.880	6.8	6.8	0.07	0.777	5.8	7.8	0.07	<0.001
d 25	10.7	10.7	0.13	0.903	10.7	10.6	0.13	0.562	9.3	12.1	0.13	<0.001
Pigs that lost BW after weaning, %^2^												
d 3	88.9	83.1	3.14	0.148	89.8	81.7	3.22	0.048	83.3	88.8	3.06	0.180
d 7	42.9	37.6	3.75	0.313	41.9	38.6	3.72	0.540	35.8	44.8	3.73	0.091
d 0 to 7												
ADG, g	3.0	15.2	4.70	0.072	6.5	11.7	4.70	0.443	12.4	5.8	4.70	0.322
ADFI, g	69	78	3.1	0.056	72	75	3.1	0.467	70	77	3.1	0.081
G:F, g/kg	−62	140	85.3	0.099	5	73	85.3	0.575	118	−40	85.3	0.195
d 7 to 14												
ADG, g	170	168	7.0	0.753	171	166	7.0	0.552	146	191	7.0	<0.001
ADFI, g	237	249	5.9	0.163	245	241	5.9	0.640	215	271	5.9	<0.001
G:F, g/kg	711	671	22.6	0.216	699	684	22.6	0.634	679	704	22.6	0.430
d 14 to 25												
ADG, g	350	352	8.1	0.831	356	346	8.1	0.374	311	392	8.1	<0.001
ADFI, g	469	474	11.8	0.793	478	465	11.8	0.449	413	530	11.8	<0.001
G:F, g/kg	749	751	13.6	0.928	752	749	13.6	0.865	757	744	13.6	0.498
d 0 to 25												
ADG, g	202	206	4.7	0.508	207	201	4.7	0.413	181	227	4.7	<0.001
ADFI, g	291	300	6.6	0.360	299	292	6.6	0.472	261	331	6.6	<0.001
G:F, g/kg	692	691	9.4	0.897	694	689	9.4	0.703	694	689	9.4	0.752

^1^For the pre-weaning portion of the experiment, a total of 28 litters (241 × 600, DNA, Columbus, NE) were used during one lactation period. Treatments consisted of a negative control (without powder) or a treatment with powder, in which approximately 45 g of sensory attractant powder were provided to litters of pigs twice daily (AM and PM) in the pan of rotary creep feeders for 4 d prior to weaning. For the post-weaning portion of the experiment, a total of 355 pigs were weaned and used in a 25-d growth trial with 5 or 6 pigs per pen and 15 replicates per treatment. Treatments were arranged in a 2 × 2 × 2 factorial with main effects of pre-weaning treatment (without or with powder); post-weaning treatment (without or with powder); and BW category (light or heavy). Pens of pigs assigned to the powder treatment group were offered approximately 22.5 g of sensory attractant powder twice daily (AM and PM), that was top-dressed on feed in feeder pans for 2 d after weaning. On d 3, approximately 22.5 g of feed (taken from the back of the feeder) was top-dressed on feed in the feeder pans twice daily to mimic powder application.

^2^Represents the percentage of pigs that were still below weaning weight 3 or 7 d post-weaning.

**Figure 2.**
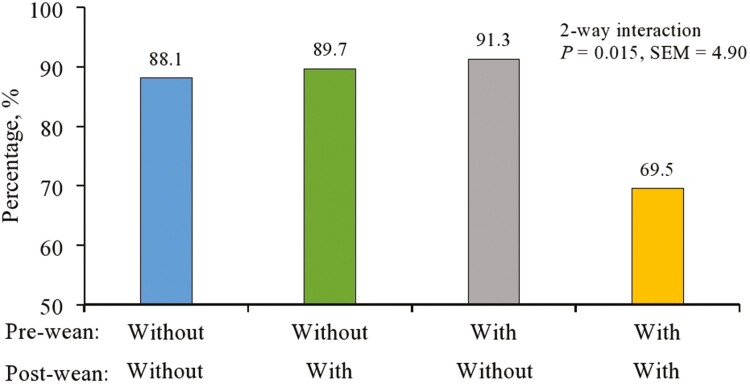
Pre-weaning sensory powder × post-weaning sensory powder interaction on the percentage of pigs that lost weight from weaning to d 3. No two-way interaction from weaning to d 7 was observed (*P* > 0.10).

For the percentage of pigs that were still below weaning weight 3 or 7 d post-weaning, no three-way interactions were observed (*P* > 0.10). An interaction between pre × post-weaning treatment was observed from weaning to d 3 (*P* *=* 0.015; Figure 2). Providing powder both pre- and post-weaning reduced the percentage of pigs that lost weight by approximately 20 percentage points compared to the other three treatment combinations. This interaction diminished by d 7 and no other interactions (*P* > 0.10) were observed. The main effects of pre- and post-weaning powder application and BW category are shown in Table 4. Significance was detected on d 3 for the main effect of post-weaning treatment (*P* = 0.048); however, this effect was driven by the interaction between pre × post-weaning treatment as previously described. Numerically, a greater percentage of heavy weight pigs lost weight (*P* = 0.091) during the first 7 days post-weaning compared to the lightweight pigs, which may support the numerically reduced ADG in heavy weight pigs observed from d 0 to 7 post-weaning.

### Experiment 3

At the start of the experiment, piglet 24-h birth weight was significantly heavier (*P* *=* 0.026) for litters assigned to the liquid sensory attractant treatment compared to litters assigned to the control group. This was a result of the randomized allotment procedure over the 5-d farrowing window. However, no differences were observed in total piglet gain pre-weaning (*P* > 0.10). Therefore, litters that received liquid sensory attractant during lactation continued to have numerically heavier BW at weaning compared to litters that did not receive liquid sensory attractant. However, this was not statistically significant (*P* > 0.10).

No three-way interactions between pre- and post-weaning treatment and BW category (*P* > 0.10) were observed for growth performance after weaning (data not shown). Likewise, no 2-way interactions (*P* > 0.10) between pre × post-weaning treatment were observed, except for BW on d 0 (*P* = 0.005), which reflects the differences in starting BW (24-h birth) as previously discussed. No evidence for treatment differences (*P* > 0.05) were observed for the interaction between post-weaning treatment × BW category; however, interactions between pre-weaning treatment × BW category were observed (Table 5; interactive means are only shown for the significant response variables). Application of a liquid sensory attractant pre-weaning had no effect on the ADG of light weight pigs from d 14 to 24 and 0 to 24, whereas in the heavy weight pig population, pre-weaning application increased gain (*P* < 0.01). This was driven by differences in ADFI (*P* < 0.05). No differences in G:F were observed (*P* > 0.10).

**Table 5.** Body weight category × pre-weaning sensory attractant liquid interaction on the growth performance of nursery pigs, Exp. 3^1^

Item	BW category				SEM	*P* = ^2^ Interaction
	Light		Heavy			
	Pre-wean liquid					
	Without	With	Without	With		
Post-wean BW, kg						
d 24	10.5^c^	10.2^c^	13.1^b^	13.8^a^	0.17	0.008
d 14 to 24						
ADG, g	388^c^	366^c^	436^b^	474^a^	10.1	0.004
ADFI, g	534^c^	515^c^	614^b^	657^a^	12.1	0.013
d 0 to 24						
ADG, g	233^c^	217^c^	260^b^	283^a^	6.7	0.005
ADFI, g	335^b^	314^b^	382^a^	402^a^	8.1	0.013

^a,b,c^Means lacking common superscript differ, *P* < 0.05.

^1^For the pre-weaning portion of the experiment, a total of 28 litters (241 × 600, DNA, Columbus, NE) were used during one lactation period. Treatments consisted of a negative control (without liquid) or a treatment with liquid, in which approximately 3 fluid ounces of liquid sensory attractant per day, divided into 2 applications (AM and PM), were sprayed on the underline of sows for 2 d beginning the morning after farrowing. Treatment application was later resumed for 2 d prior to weaning. In total, pigs received liquid sensory attractant for 4 d pre-weaning. For the post-weaning portion of the experiment, a total of 355 pigs were weaned and used in a 24-d growth trial with 5 or 6 pigs per pen and 15 replicates per liquid attractant treatment. Treatments were arranged in a 2 × 2 × 2 factorial with main effects of pre-weaning treatment (without or with liquid); post-weaning treatment (without or with liquid); and BW category (light or heavy). Pens of pigs assigned to the liquid attractant treatment group were offered approximately 2 fluid ounces of liquid per day, divided into 3 applications (7:00, 12:00, and 17:00), sprayed on the feed. Treatment application continued for 3 d post-weaning.

^2^No three-way interactions between pre- and post-weaning treatment and BW category were observed for growth performance after weaning (*P* > 0.10). Likewise, no post-weaning treatment × BW category interactions were observed (*P* > 0.10). For ease of interpretation, only the significant response variables are shown for the interactions between pre-weaning treatment × BW category.

Based on how pigs were then allotted to pens post-weaning, a statistical difference for the main effect of pre-weaning liquid application was observed for d 0 BW (*P* < 0.001). Again, this was a result of lighter weights (24-h birth) at the start of the experiment for pigs that did not receive liquid attractant before weaning. No other differences (*P* > 0.05) were observed for the main effect of pre-weaning attractant thereafter. Likewise, no differences (*P* > 0.10) for the main effect of post-weaning attractant were observed. Treatment differences were observed for the main effect of BW category. Overall (d 0 to 24), heavy weight pigs had increased ADG (*P* *<* 0.001) and ADFI (*P* *<* 0.001) compared to light weight pigs. No differences G:F were observed (*P* > 0.10).

**Figure 3.**
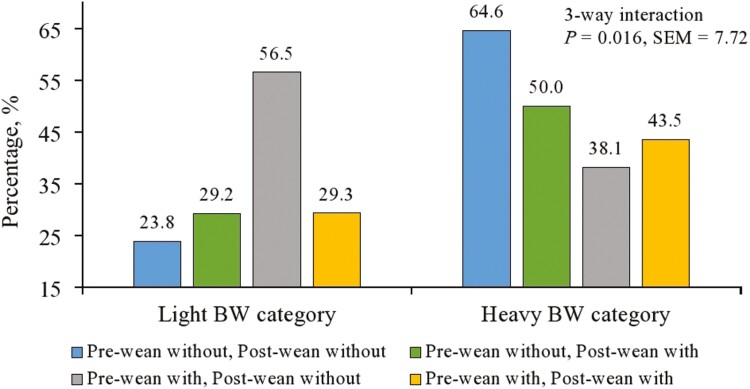
Body weight category × pre-weaning sensory liquid × post-weaning sensory liquid interaction on the percentage of pigs that lost weight from weaning to d 3. No three-way interaction from weaning to d 7 was observed (*P* > 0.10).

For the percentage of pigs that were still below weaning weight 3 or 7 d post-weaning, a three-way interaction was observed from weaning to d 3 (Figure 3; *P* *=* 0.016). A greater percentage of heavy weight pigs that did not receive liquid sensory attractant lost weight compared the other three treatment combinations. In contrast, a lower percentage of light weight pigs that received liquid sensory attractant either not at all, only post-weaning, or both pre- and post-weaning lost weight compared to pigs that received liquid sensory attractant only pre-weaning. The interaction observed diminished by d 7 (*P* > 0.10) but does indicate a difference in how light and heavy weight pigs may respond to sensory intervention strategies. The main effects of liquid attractant application can be found in Table 6; however, because the three-way interaction on d 3 was already discussed, the main effects will not be emphasized. From weaning to d 7 post-weaning, a greater percentage of heavy weight pigs lost weight than light weight pigs (*P* *=* 0.051), which agrees with the results of Exp. 1 and 2.

**Table 6.** Main effect of pre-weaning sensory attractant liquid, post-weaning sensory attractant liquid, and BW category on the growth performance of nursery pigs, Exp. 3^1^

Item	Pre-wean liquid		SEM	*P* =	Post-wean liquid		SEM	*P* =	BW category		SEM	*P* =
	Without	With			Without	With			Light	Heavy		
Pre-wean BW, kg												
24-h birth	1.4	1.6	0.05	0.026								
Weaning	5.8	6.0	0.18	0.559								
Total gain, g	4,423	4,403	164.6	0.934								
Average litter size, n	15.4	13.8	0.75	0.151								
Post-wean BW, kg												
d 0	5.8	5.9	0.02	< 0.001	5.9	5.9	0.02	0.141	4.9	6.9	0.02	< 0.001
d 3	5.8	5.9	0.05	0.122	5.8	5.9	0.05	0.126	4.9	6.8	0.05	< 0.001
d 7	6.3	6.3	0.06	0.419	6.3	6.3	0.06	0.493	5.3	7.3	0.06	< 0.001
d 14	7.7	7.8	0.07	0.189	7.7	7.8	0.07	0.549	6.6	8.9	0.07	< 0.001
d 24	11.8	12.0	0.12	0.213	11.9	11.9	0.12	0.992	10.4	13.4	0.12	< 0.001
Pigs that lost BW after weaning, %^2^												
d 3	41.0	41.5	3.91	0.928	45.1	37.6	3.97	0.173	33.9	49.1	3.83	0.007
d 7	9.4	16.3	2.89	0.075	12.2	12.6	2.81	0.909	9.1	16.7	2.87	0.051
d 0 to 7												
ADG, g	62	54	7.6	0.493	58	58	7.6	0.998	63	53	7.6	0.342
ADFI, g	160	151	6.6	0.349	152	159	6.6	0.479	146	165	6.6	0.050
G:F, g/kg	375	305	46.1	0.286	343	338	46.1	0.939	392	289	46.1	0.119
d 7 to 14												
ADG, g	197	206	6.0	0.287	199	203	6.0	0.666	175	228	6.0	< 0.001
ADFI, g	253	245	6.7	0.363	247	251	6.7	0.724	226	272	6.7	< 0.001
G:F, g/kg	783	845	24.1	0.076	809	819	24.1	0.772	784	844	24.1	0.084
d 14 to 24												
ADG, g	412	420	7.2	0.448	419	413	7.2	0.538	377	455	7.2	< 0.001
ADFI, g	574	586	8.6	0.330	580	579	8.6	0.948	524	635	8.6	< 0.001
G:F, g/kg	718	716	6.3	0.820	723	712	6.3	0.231	719	716	6.3	0.789
d 0 to 24												
ADG, g	246	250	4.7	0.622	249	248	4.7	0.882	225	271	4.7	< 0.001
ADFI, g	359	358	5.7	0.946	357	360	5.7	0.677	325	392	5.7	< 0.001
G:F, g/kg	687	695	7.0	0.422	696	687	7.0	0.389	691	691	7.0	0.993

^1^For the pre-weaning portion of the experiment, a total of 28 litters (241 × 600, DNA, Columbus, NE) were used during one lactation period. Treatments consisted of a negative control (without liquid) or a treatment with liquid, in which approximately 3 fluid ounces of liquid sensory attractant per day, divided into 2 applications (AM and PM), were sprayed on the underline of sows for 2 d beginning the morning after farrowing. Treatment application was later resumed for 2 d prior to weaning. In total, pigs received liquid sensory attractant for 4 d pre-weaning. For the post-weaning portion of the experiment, a total of 355 pigs were weaned and used in a 24-d growth trial with 5 or 6 pigs per pen and 15 replicates per liquid attractant treatment. Treatments were arranged in a 2 × 2 × 2 factorial with main effects of pre-weaning treatment (without or with liquid); post-weaning treatment (without or with liquid); and BW category (light or heavy). Pens of pigs assigned to the liquid attractant treatment group were offered approximately 2 fluid ounces of liquid per day, divided into three applications (7:00, 12:00, and 17:00), sprayed on the feed. Treatment application continued for 3-d post-weaning.

^2^Represents the percentage of pigs that were still below weaning weight 3 or 7 d post-weaning.

## DISCUSSION

The environment in which pigs are reared affects their behavioral development (Lau et al., 2015). Pigs raised outdoors have been identified as calm/passive and spend more time eating post-weaning, whereas pigs raised indoors have been identified as playful/investigative and spend more time exploring their environment (Lau et al., 2015). While investigation is key to getting pigs started on feed, providing indoor reared pigs early opportunities to develop feeding behaviors through sensory enrichment, may lead to early feed intake post-weaning. Oostindjer et al. (2011a) observed that first introducing a novel food type to young pigs in the presence of the sow reduced latency to touching feed and improved feed intake compared to first introducing a novel food type in the absence of the sow. This suggests that piglets are more comfortable first interacting with feed when in a familiar environment. In contrast, if a novel food was first introduced in the absence of the sow, consumption was improved if piglets had environmental enrichment. This suggests that providing environmental stimuli when pigs are not in a familiar environment may stimulate the pigs natural play/foraging behavior, therefore increasing pen exploration. Because weanling pigs are sensitive to sensory factors including, taste, odor, and texture, and are highly motivated by environmental stimuli (Sola-Oriol et al., 2009; Oostindejer et al., 2011b), providing environmental or flavor enrichment during multiple phases of early life, when piglets are in a familiar environment, may help stimulate behavioral development and reduce stress when that same enrichment is provided post-weaning (Oostindejer et al., 2014). Therefore, feed intake may be increased, reducing the post-weaning growth check.

Creep feeding is another strategy that can be used to develop feeding behaviors. Providing creep feed to pigs during lactation encourages exploratory behavior and familiarizes pigs with solid feed before weaning; consequently, improving feed intake and BW gain in the immediate post-weaning period (Bruininx et al., 2002). More recently, Middelkoop et al. (2019) observed that providing creep feed in play feeders (conventional rotary feeders with canvas cloth, braided cotton ropes, and PVC spiral tubes attached on the feeder) elicited play behavior, attracting more pigs to creep feed. This response followed pigs through the immediate post-weaning period where increased feed intake and gain were observed. The authors suggested that providing creep feed in play feeders prior to weaning may develop a positive association between solid feed and object play, stimulating greater feed consumption.

Similar learning benefits to creep feeding have also been explored in other forms such as environmental and flavor stimuli. The goal of both strategies is to stimulate investigative activity and help pigs overcome fear of their new environment, therefore leading to increased feed intake. For this reason, we used three strategies to provide pre-weaning enrichment that continued through to the post-weaning period, including large enrichment cubes (Exp. 1), a powder attractant (Exp. 2), and a liquid spray attractant (Exp. 3). Environmental enrichment (straw, wood shavings, peat, and branches) pre-weaning has been shown to increase the floor exploration and play behavior of pigs prior to weaning. However, these behaviors did not follow pigs into the nursery unless environmental enrichment was also offered post-weaning (Oostindejer et al., 2011b). Interestingly, when looking at growth performance effects, Oostindjer et al. (2010b) observed that pre-weaning enrichment alone increased feed intake in the first 48 h after weaning. This response may be related to the development of foraging behaviors or decreased stress. Similar to the pre-weaning behavioral response, post-weaning enrichment alone has been shown to increase pen and floor exploration, play behavior and general activity (Oostindjer et al., 2011b). In contrast to the pre-weaning growth performance response, this led to enriched pigs exhibiting decreased feeder interactions and no differences in feed intake compared to pigs that were not enriched after weaning (Oostindjer et al., 2011b). However, in an earlier experiment, Oostindjer et al. (2010b) observed that post-weaning enrichment alone increased the growth of pigs during the first 14 d in the nursery. While it appears enrichment consistently impacts pig behavior, the growth performance benefits are equivocal.

In Exp. 1, providing large enrichment cubes before weaning, but not after, led to decreased feed intake and BW gain, with the greatest response observed when heavy weight pigs were provided cubes both pre- and post-weaning or not at all. In contrast, light weight pigs provided enrichment cubes before weaning, but not after, exhibited increased feed intake and BW gain. Despite the variation seen in growth performance between light and heavy weight populations, the addition of large enrichment cubes only post-weaning reduced the percentage of pigs that lost weight after weaning. Likewise, a greater percentage of pigs lost weight when offered enrichment cubes only pre-weaning. This response may be related to loss of enrichment at weaning. These data in combination with Oostindjer et al. (2010b, 2011b) suggest that post-weaning environmental enrichment may provide the greatest opportunity to improve the weaning transition.

Pre- and postnatal flavor exposure through the maternal diet has also been suggested to reduce feed neophobia post-weaning (Oostindjer et al., 2010a). However, the data appears to be more consistent for prenatal exposure than postnatal. Figueroa et al. (2013) showed that prenatal exposure to anise or milky cheese through the maternal diet increased pig preference for the previously exposed flavor up to 2 d post-weaning. This may indicate that preferences acquired prenatally can be long-lasting. However, it is important to note that flavor preference in behavioral studies does not always equate to improved growth performance. In two separate studies, prenatal flavor exposure did not increase the feed intake of flavored creep feed during the suckling period compared to unflavored creep feed (Figueroa et al., 2013; Blavi et al., 2016). Varying responses have also been reported post-weaning. Oostindjer et al. (2010a) showed that prenatal exposure to anise increased pig BW up to 5 d post-weaning compared to pigs that were not exposed prenatally. However, no differences in performance were observed with postnatal exposure alone or the interaction between pre- and postnatal exposure. In contrast, Blavi et al. (2016) showed that feeding sows a flavor compound of anethol, cinnamaldehyde, and eugenol in late gestation through lactation increased piglet feed intake post-weaning, regardless of post-weaning diet. However, this did not lead to improvements in gain. Oostindjer et al. (2011c) also assessed the effect of prenatal flavor exposure on weaning stress. The authors observed a reduction in saliva cortisol levels 4 h post-weaning when pigs were exposed to flavors prenatally and then re-introduced to that same flavor after weaning. Likewise, latency to eat was decreased, play behavior increased, and both vocalization and manipulation behaviors decreased. Unfortunately, this did not result in increased feed intake or gain. These data may indicate that pigs prefer the scent of previously exposed flavors, rather than taste, which may explain the inconsistent response of postnatal flavor exposure observed throughout the literature (Oostindjer et al., 2011c). Likely, this is a result of a flavor association between olfactory stimuli and positive life experiences, suggesting that flavor learning works by reducing stress due to the presence of a familiar smell, rather than something that tastes good. Unfortunately, this does not consistently translate into improved performance after weaning. Compared to environmental enrichment, it appears that prenatal flavor exposure that continues through lactation is a strategy that may provide opportunities to improve the weaning transition (Oostindejer et al., 2014). Although limited differences in growth performance were observed for Exp. 2 and 3, the percent of pigs that lost BW after weaning agrees with the previous literature. In Exp. 2, sensory attractant powder exposure both pre- and post-weaning reduced the percentage of pigs that lost weight compared to when exposure occurred only pre-weaning, only post-weaning, or not at all. In Exp. 3, exposure to a liquid sensory attractant pre-weaning, post-weaning, or both pre- and post-weaning decreased the percentage of heavy weight pigs that lost weight compared to no exposure. While the response to liquid sensory attractant exposure among light weight pigs was variable, the overall percentage of light weight pigs that lost weight from d 0 to 3 post-weaning was lower than the percentage of heavy weight pigs. The results herein further emphasize the variable responses observed in sensory learning trials.

Most behavioral studies that assess flavor or environmental enrichment look at piglet preference rather than BW loss. Hence, it’s challenging to compare the results of these experiments with previous reports. Based on the studies herein, it does appear sensory attractants have the potential to reduce the percentage of pigs that lose weight immediately post-weaning. The effect of sensory attractant application on BW loss post-weaning also seems to be related to piglet BW at weaning, with a greater percentage of heavy weight pigs exhibiting BW loss initially post-weaning compared to light weight pigs. While limited differences in ADG and ADFI were observed from d 0 to 7 post-weaning for the main effect of BW category across all three experiments, reductions in the percentage of light weight pigs that lost weight may be a result of initial feed intake. Bruininx et al. (2001) showed that feed intake during the first 24-h post-weaning was increased in light weight pigs compared to heavy pigs. This continued for the first 3 d post-weaning, indicating that heavy weight pigs take longer to begin consuming feed, particularly when sorted into pens of all heavy weight pigs (Bruininx et al., 2001), which was the case in these experiments. Because heavy pigs are often more dominant, it is likely that heavy pigs spend more time engaged in aggressive interactions to establish social hierarchy than searching out and consuming feed (Faccin et al., 2019). A second factor influencing the greater number of heavy pigs that lose weight immediately post-weaning is the possibility that heavy weight pigs do not seem to have to compete for feed resources while on the sow compared to light weight pigs. Therefore, they might not have explored alternative feed options like creep feed. Despite greater initial BW loss, the overall ADG and feed intake of heavy weight pigs was increased compared to light weight pigs, which agrees with Bruininx et al. (2001). Together, these data emphasize that all pigs, regardless of starting BW, struggle to make the weaning transition.

In summary, sensory attractant application had limited effects on the growth performance of pigs after weaning; however, varying responses were observed for the percentage of pigs that lost weight immediately post-weaning, as measured by the percentage of pigs that were still below weaning weight 3 or 7 d post-weaning. Environmental enrichment with cubes (Exp. 1) appears to have the greatest effect when applied post-weaning whereas flavor attractants (Exp. 2 and 3) appear to have the greatest effect when applied both pre- and post-weaning. Piglet BW at weaning also plays an important role in behavioral development and their subsequent response to sensory attractants. Strategies to reduce the number of pigs that lose weight after weaning warrant further investigation, with an emphasis on understanding how BW loss post-weaning impacts survival and lifetime gain.

## Abbreviations

ADG: average daily gain
ADFI: average daily feed intake
BW: body weight
G:F: gain-to-feed ratio
